# Intergenerational Transmission of Anxious Information Processing Biases: An Updated Conceptual Model

**DOI:** 10.1007/s10567-022-00390-8

**Published:** 2022-02-26

**Authors:** Evin Aktar

**Affiliations:** 1 Department of Psychology, Clinical Psychology Unit, Leiden, The Netherlands; 2 Leiden Institute for Brain and Cognition, Leiden University, Leiden, The Netherlands

**Keywords:** Cognition, Parenting, Anxiety, Attention bias, Threat interpretations

## Abstract

Anxiety disorders are globally one of the most prevalent and disabling forms of psychopathology in adults and children. Having a parent with an anxiety disorder multiplies the risk of anxiety disorders in the offspring, although the specific mechanisms and processes that play a role in this intergenerational transmission remain largely unknown. According to information processing theories, threat-related biases in cognitive processing are a causal mechanism in the development and maintenance of anxiety. These theories propose that individuals with anxiety are more likely to cognitively process novel stimuli in their environment as threatening. Creswell and colleagues proposed a theoretical model that highlighted the role of these cognitive biases as a mechanism in the intergenerational transmission of anxiety (Creswell et al., in Hadwin, Field (eds) Information processing biases and anxiety: a developmental perspective, Wiley, pp 279–295, 2010). This model postulated significant associations between (1) parents’ and children’s threat-related cognitive biases (2) parents’ threat-related cognitive biases in their own and their child’s environment, (3) parents’ threat-related cognitive biases and parenting behaviors that convey anxiety risk to the offspring (e.g., modeling of fear, and verbal threat information transmission), and (4) parenting behaviors and child threat-related biases. This theoretical review collated the recent empirical work testing these four core hypotheses of the model. Building on the reviewed empirical work, an updated conceptual model focusing on threat-related attention and interpretation is proposed. This updated model incorporates the links between cognition and anxiety in parents and children and addresses the potential bidirectional nature of parent–child influences.

Anxiety disorders are among the most prevalent and disabling forms of psychopathology in adults and children across the globe (Baxter et al., 2013; Polanczyk et al., 2015). As such, anxiety disorders globally pose a financial and psychological burden to society, families, and individuals (Baxter et al., 2014). Evidence from family studies shows that anxiety runs in families: Having a parent with anxiety disorder multiplies the risk of anxiety disorder in the offspring (Adolph et al., 2020; Lawrence et al., 2019; Telman et al., 2018). Estimated genetic heritability across anxiety disorders reveals a modest but significant contribution of genetic transmission, with the remaining variance explained by environmental influences and gene*environment interplays (Eley et al., 2015; Hettema et al., 2001). Despite growing evidence for familial aggregation of anxiety, there is limited knowledge on the specific non-genetic mechanisms and processes that play a role in its emergence. A better understanding of family processes that contribute to familial aggregation is crucial for the prevention and intervention efforts aiming to break intergenerational anxiety transmission.

Information processing theories of anxiety indicate that biases in cognitive processes are causally related to the development and maintenance of anxiety in children and adults (Beck & Clark, 1997; Mogg & Bradley, 1998). These cognitive biases are marked by a higher likelihood to process novel/ambiguous stimuli as threatening across the stages of information processing (e.g., attention, interpretation, reasoning, and memory). There is empirical evidence supporting the view that these threat-related biases characterize the information processing of anxious youth and adults: anxious individuals are not only faster to detect and orient towards potentially threatening stimuli in the early stages of information processing (Bar-Haim et al., 2007; Dudeney et al., 2015; Van Bockstaele et al., 2014), but they also are more likely to interpret or perceive novel stimuli as threatening (Chen et al., 2020; Stuijfzand et al., 2018) at later processing stages.

Building on this evidence of a causal role of cognitive biases in anxiety, Creswell and colleagues proposed a theoretical model on the role of information processing biases as a mechanism in the intergenerational transmission of anxiety (Creswell et al., 2010). This model, presented in Fig. 1, consisted of four core hypotheses. First, similarities were postulated between parent and child cognition (‘1’ in Fig. 1), referring to a positive association between the information processing styles of parents and those of their offspring. As such, children of anxious parents who show stronger threat-related biases when processing novel stimuli would also show stronger threat-related biases. Second, threat-related biases in parents’ information processing were proposed to extend to the child’s environment, and to parents’ expectations of their child’s perceptions of coping with novelty (‘2’ in Fig. 1). This implies that threat-related biases in anxious parents’ information processing of novelty in their environment will apply similarly to potential threats in their child’s environment. Thus, for example, anxious parents would be more likely to interpret novel situations as threatening for their child and to expect their child to do the same. Third, parents’ information processing style was proposed to be related to specific parenting behaviors that convey anxiety to the offspring (‘3’ in Fig. 1). Thus, threat-related biases in anxious parents’ information processing would be related to a higher likelihood of engaging in fear-enhancing parenting (for example, modeling of non-verbal signals of anxiety, and verbal threat information transmission) and in a lower likelihood of autonomy granting (for example, parental overcontrol and overinvolvement). Fourth, these parenting behaviors were proposed to be, in turn, linked to the child’s information processing biases (‘4’ in Fig. 1). Therefore, children whose parents are more likely to show fear-enhancing behaviors, or overcontrolling parenting, would show stronger biases towards threat in their processing.

**Fig. 1.**
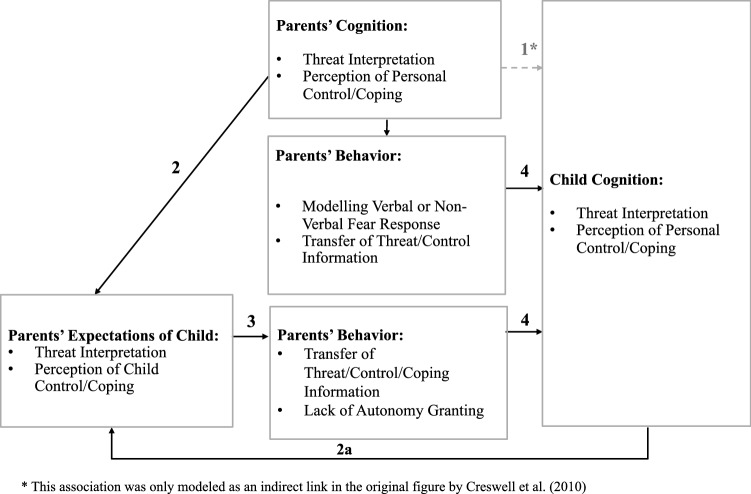
The cognitive-behavioral model of intergenerational transmission of anxious information processing biases (Reprinted with permission from Creswell et al., 2010)

Twelve years on from the publication of this model, this theoretical review aims to revisit these core hypotheses on the intergenerational transmission of anxious information processing (Creswell et al., 2010) by highlighting the recent empirical work on each of the proposed associations. As it will shortly become clear, the recent work is marked by a considerable variety in the study samples, designs, and methods, as well as in the operationalization of cognition and parenting constructs. This variety made it non-viable to present a systematic and elaborate description of all empirical work on each of the hypotheses while maintaining the current emphasis on the model as a whole. Instead, this review paper aims to explore the empirical studies published in the period between 2010 and mid-2021 that cited this model and/or tested one or more of the hypothesized associations. A description of the recent systematic reviews and/or meta-analytic work on the specific associations were included whenever available (e.g., Subar & Rozenman, 2021, on the association between parent and child interpretation biases, or Emerson et al., 2019, on the parental pathways to child anxious cognitions).

In the sections that follow, a narrative overview of the recent evidence of each of the four hypotheses is presented, first with threat-related interpretation and then with attention. Empirical studies on alternative stages of information processing (e.g., confirmation and search bias, fear beliefs, coping/distress/threat perceptions) were incorporated per section if available. Earlier empirical studies reviewed by Creswell et al. (2010) were briefly revisited especially when there is little or no recent work on a given association. The review concludes with an updated conceptual model on the intergenerational transmission of anxious information processing biases in the light of the reviewed empirical evidence. Future research directions and clinical implications are also discussed.

## Do Parents and Children Share a Common Information Processing Style for Threat?

The model by Creswell et al. (2010) postulates similarities between the information processing styles of parents and their children, as a result of shared genetic and/or environmental influences. Thus, children of anxious parents who show stronger threat-related biases when processing novel stimuli would also show stronger threat-related biases. In the absence of earlier empirical work that tested this link at any other stages of information processing, this link was mainly constructed based on threat interpretations in the original model. Although still in its infancy, a new line of research addressing this association in attention has recently emerged. In addition, a few empirical studies have tested the direct association between parent and child cognition at the level of reasoning (i.e., confirmation and search bias) in experimental designs.

### Threat Interpretations

The first empirical studies addressing the similarities in the threat interpretations of parents and children used the ambiguous scenarios task in 7-to-15-year-old children and their parents (e.g., Creswell & O’Connor, 2006; Creswell et al., 2005). In this task, participants are presented with ambiguous scenarios depicting situations with two alternative interpretations (threatening and non-threatening) and are asked to indicate the most likely interpretation. The evidence from these earlier studies is mixed: Some studies reported significant associations between the likelihood of interpreting novel stimuli as threatening in parents and children (e.g., Creswell & O’Connor, 2006; Creswell et al., 2005), and others did not (e.g., Creswell et al., 2006; Gifford et al., 2008).

A recent systematic review and meta-analysis on the concurrent link between parent and child threat interpretations included these earlier studies in addition to four more recent studies (Subar & Rozenman, 2021, for an overview of the studies, see p. 311 Table 1). The findings provided support for the hypothesized link, but revealed only a small association between parent and child interpretation biases (*K* = *8, r* = 0.15, *p* < 0.01, Subar & Rozenman, 2021). This may be explained by the mixed findings both in earlier studies and in the more recent evidence (summarized in Subar & Rozenman, 2021). Some recent studies provided support for this link (Păsărelu et al., 2017; Podina et al., 2013) whereas others did not (Neil et al., 2019; Ooi et al., 2015). Moreover, the meta-analytic evidence for similarities in threat interpretations of parents and children must be cautiously evaluated in light of several limitations, including the relatively small number of available empirical studies, which precludes firm conclusions on this link. A further limitation was the considerable heterogeneity of the samples investigated in terms of child age and anxiety status: the age of the children varied between 4 and 17 years, and the samples were selected based on child anxiety in only some of the studies. The limited number of studies in this meta-analysis did not allow for testing potential moderators of the link between threat interpretations in parents and children (Subar & Rozenman, 2021). Thus, it currently remains unclear how this association differs as a function of age and anxiety status. Another limitation concerns the variability of the tasks used in the measurement of interpretation biases in parents and children (e.g., Gifford et al., 2008; Ooi, 2015). Moreover, the extent to which the interpretation biases captured in these tasks relate to parents’ and children’s interpretation of novel/ambiguous situations in real-life is unclear. A final limitation concerns the cross-sectional nature of the designs, precluding any inferences regarding the transmission of interpretation bias from parents to children.

**Table 1:** Studies testing the association between parent and child attention to threat

Study(author last name, et al., year)	Youth *N Gender* (% female)	Youth Age *M (SD, range)*	Target Population	Youth Anxiety Symptom Measure	Youth Threat Bias Measure	Parent *N Gender* (% female)	Parent Psychopathology	Parent Threat Bias Measure	Link Parent–Child Bias	P-value
Mogg et al. (2012)	57 (100)	11.7 (not reported, 9–14)	Daughters of mothers diagnosed with panic disorder	STAI-C	Visual Probe Task with threat vs neutral words and pictures	57(100)	Panic disorder	Visual Probe Task with threat vs neutral words and pictures	-.16 < *r* < .16	> .2
Waters et al. (2015)*	67 (61)	9.4 (1.4, not reported) in low risk and 9.3 (1.6, not reported) in high-risk groups	Children at high versus low risk (based on the maternal diagnoses of emotional disorder)	ADIS-C-IV Spence Children's Anxiety Scale (parent and child report)	Visual Probe Task with threat (angry) versus neutral faces	67(100)	Depression and anxiety diagnosis (versus no diagnosis)	Visual Probe Task with threat (angry) vs neutral face pairs	-29 < *r* < .19*	> .1
Waters et al. (2018)	43 (58)	9.7 (1.3, 7–12)	Children at high versus low risk (based on the maternal diagnoses of emotional disorder)	ADIS-C-IV	Visual Probe Task with threat (angry) versus neutral faces	43 (100)	Depression and anxiety diagnosis (versus no diagnosis)	Visual Probe Task with threat (angry) vs neutral face pairs	-.14 ≤ *r* ≤ .00	> .05
Aktar et al. (2019)	89 (52.8)	7.5 (0.1, not reported)	Unselected	ADIS-P-IV, SCARED‐P	Visual Probe and Visual Search Tasks with threat (angry) versus happy faces	198 (50)	GAD and SAD symptoms	Visual Probe and Visual Search Tasks with threat (angry) versus happy faces	−.14 ≤ *r* ≤ .16	> .05

M = Mean, SD = standard deviation, r: correlation, b: unstandardized estimate, SE: Standard Error

^*^The correlation between negative attention bias of parents and children was not separately reported in this study. The range of reported correlations includes the link between negative attention bias scores of parents and children, and symptom measures of depression and anxiety

A later longitudinal study (Creswell et al., 2011) investigating threat interpretations of 5-to-9-year-old children and their parents in three successive yearly measurements did not support the idea that stronger threat interpretations in parents would predict stronger child threat interpretations, either cross-sectionally or longitudinally. Taken together, the findings highlight the need to establish the developmental emergence and the timeline of the association between interpretation biases of parents and children. It also remains to be explored whether the strength of commonalities differs as a function of age and anxiety status.

### Attention to Threat

A recent line of research focuses on the link between parental and child attention biases (Aktar et al., 2019; Mogg et al., 2012; Waters et al., 2015, 2018 see Table 1 for an overview). These studies have predominantly relied on the visual probe task to measure attention bias. In this task, a threat relevant and a non-threat relevant stimulus appear side by side on the screen, followed by a probe. The subjects are instructed to respond to the location of the probe with a button press. An attentional bias to threat is inferred when participants react faster on trials where the probe appears on the location of the threat relevant stimuli (congruent trials) versus trials where the probe appears on the location of the non-threat relevant stimuli (incongruent trials).

The use of the visual probe task is a major limitation characterizing this line of research as the task can yield unreliable attention bias scores in adult and child populations (Brown et al., 2013; Schmukle, 2005) and has been deemed less suitable for the investigation of individual differences (De Schryver et al., 2016; Schmukle, 2005). There are also some methodological differences between studies in the computation of threat-related bias in attention: Some studies have utilized positive stimuli whereas others use neutral stimuli as a reference for threat-related stimuli. Other limitations that characterize the empirical work on threat interpretations also apply to attention: there is heterogeneity in the samples investigated with respect to age and parental/child diagnostic status.

The first empirical study investigating parent and child attention to threat (versus neutral stimuli) using the visual probe task relied on a clinical sample of mothers with (versus without) lifetime panic disorder and their 9-to-14-year-old daughters (Mogg et al., 2012). This study did not reveal a significant link between mothers’ and daughters’ attention biases to threat. The second study investigated attention biases in a similar visual probe task with angry versus neutral faces in children and their mothers (with versus without emotional disorder, Waters et al., 2015). In addition to the negative attention bias scores computed as the differential reaction time to angry versus neutral faces, this study incorporated a positive bias score computed as the differential reaction time to happy versus neutral faces. The link between mothers’ and children’s negative attention biases was not significant. Interestingly, however, among high-risk children (due to the presence of emotional disorders in the mother), there was a negative association between child negative attention bias and maternal positive attention bias. High-risk children demonstrated a negative attention bias only if their mother did not show a positive attention bias, suggesting that a positive attention bias in diagnosed parents may act as a buffer against the development of threat-related attention bias in their children.

A follow-up analysis relied on a longitudinal design of participants in the initial assessment (Waters et al., 2015) and at a 12-month follow-up (Waters et al., 2018) to examine the link between attention bias of 7-to-12-year-old children and their parents (with versus without an emotional disorder). None of the cross-sectional or longitudinal associations between parent attention biases at T1 and child attention biases at T1 or T2 were significant in this study, irrespective of child risk status (See Table 1). Interestingly, there was a significant association between child attention bias in the first measurement and parent attention bias in the second measurement. This finding suggests a child-to-parent effect, hinting at the possible bidirectionality of the relationship between parent and child attention biases. Another study investigated attention bias to threat in parents and 7.5-year-olds using a visual probe and a visual search task with angry versus happy faces, along with anxiety symptoms of parents and children at 4.5 (T1) and 7.5 (T2) years of age (Aktar et al., 2019). Parents and children with higher levels of anxiety at T1 showed a stronger attention bias to threat at T2, whereas the study found no significant concurrent link between parents’ and the offspring’s attention biases at T2.

To summarize, the findings consistently suggest no significant links between parents’ and children’s attention biases to threat measured in the visual probe tasks, raising the question of whether the low reliability of RT measures used in these tasks makes it harder to capture this link between parent and child attention biases towards threat. More recent studies focusing on attention bias-anxiety links have implemented more reliable and ecologically valid measures of attention bias to overcome this limitation. For example, child attention bias was assessed with eye-tracking during free viewing tasks (measured with dwell times and the latency of the first fixation, see for example Shechner et al., 2013). Moreover, mobile eye-tracking technologies have been recently implemented in the study of youth cognition, allowing the measurement of attention during the child’s actual confrontations with novelty, enhancing ecological validity (Allen et al., 2020; Gunther et al., 2021). Finally, neural correlates of attention biases were explored in the visual probe task using event-related potentials (ERPs, Thai et al., 2016). Future work on threat-related attention biases among parents and children should move towards these more reliable and ecologically valid measures to reach conclusions regarding the similarities between parent and child threat-related attention.

### Search for Threat and Confirmation of Threat

Two studies examined the direct link between information processing among parents and children at the level of reasoning bias. The first study focused on confirmation bias, which refers to the likelihood of seeking for falsifying threat information about novel stimuli (Remmerswaal et al., 2010), whereas the second study focused on negative search bias (Remmerswaal et al., 2016), which refer to one’s tendency to search for verbal threat information about a novel stimulus. In the first study, researchers induced a parental confirmation bias by providing the parents with verbal threat (versus positive) information about new animals (Remmerswaal et al., 2010). Parents who received threat (versus positive) information about the animal from the experimenters showed a threat confirmation bias. That is, they were less likely to seek for information to falsify the threat value for the animals that were paired with threat information, which triggered, in turn, a threat confirmation bias towards that animal in their 9-to-12-year-old children.

The second study investigated the links between maternal (instructed or spontaneous) and child negative search bias in a sample of 8-to-13-year-olds in two experiments. In the first experiment, child search bias about novel animals was measured before and after children listened to the descriptions of the novel animals from their parents who were instructed to display a negative (versus positive) search bias about these animals during an information search task (Remmerswaal et al., 2016). The findings of the first experiment revealed that being exposed to parental positive or negative search biases induced a parallel increase in the corresponding search bias in their offspring. In the second experiment, the researchers investigated the effect of parents’ spontaneous search bias on 9-to-12-year-old children’s search bias in a similar design. The findings revealed that mothers’ initial cognitive bias scores were predictive of child cognition, such that children who were exposed to mothers’ positive or negative search bias also showed a parallel search bias. Taken together the findings from these studies suggest that a direct association between parent and child cognitions may be captured in designs targeting search for threat and confirmation of threat in late childhood years.

## Summary and Future Directions

Empirical studies on the association between parent and child threat interpretations have revealed mixed findings, whereas the available meta-analytic evidence has revealed a small but significant association between parent and child interpretation biases. In contrast, the studies on the association between parent and child attention to threat consistently showed non-significant links in childhood years. Thus, there is limited support for the association between parent and child processing of threat at this earlier stage of information processing. The few empirical studies focusing on the links between parent and child processing of threat at the stages of search and confirmation biases reveal a significant effect of parental bias on child bias. Note, however, that these conclusions remain largely preliminary today, given the few empirical studies testing the link between parent and child information processing.

Future research should adopt a developmental approach to the investigation of commonalities in parents’ and child information processing by testing the hypothesized associations in longitudinal designs from early to late childhood years. This would allow the establishment of a timeline and will provide further insight into the potential bidirectionality of the hypothesized association in parents’ and children’s information processing. Likewise, the developmental mechanisms explaining this link await further investigation. We do not yet know when a link between information processing in parents and children would emerge in development, and which genetic and/or environmental influences would account for its emergence. Furthermore, future longitudinal studies that incorporate community and clinical samples of parents (and/or children) are needed to investigate a potential moderation of this link by anxiety diagnosis. Moreover, in view of the current variability in instruments used to measure anxiety and information processing biases, future empirical studies should consider integrating two or more of these instruments in a single design. Ultimately, it will be important to consider the effects of the current variability in the sample characteristics, in the instruments, and the operationalization of the constructs of anxiety and the biases in information processing in future meta-analytic studies.

Another important question that awaits further investigation is whether the hypothesized association may be more clearly captured across different stages of information processing of parents and children, rather than at corresponding stages. De Lijster and colleagues measured attention biases of mothers, fathers, and their 8-to-17-year-old children in the visual probe paradigm with threatening versus neutral faces and child interpretations of threat using ambiguous scenarios task in a single design (de Lijster et al., 2020). In line with the earlier evidence, no direct link was observed between parent and child attention biases in this study, whereas there was a significant link between maternal attention bias to threat and child threat interpretations. Children of parents who showed a stronger attention bias to threat had a stronger bias in their threat interpretations. These findings reveal the intriguing possibility that mothers’ attentional vigilance to stimuli that signal threat may trigger child cognitive bias at later stages of information processing. Recent empirical work suggests that an integrated measurement of child cognition at the levels of attention and interpretation is possible with the help of mobile eye-tracking technologies implemented in realistic lab situations (Allen et al., 2020; Gunther et al., 2021). This option awaits to be explored in future work in the context of parent–child transmission of anxious information processing.

## Does the Information Processing Style of Parents Extend to The Threats in the Child’s Environment and Expectations of Their Child’s Cognitions?

The model by Creswell and colleagues (2010) postulates associations between parents’ prioritized processing of threat in their own and their child’s environment, and between parents’ prioritized processing of threat and parents’ expectations of their child’s threat cognitions in ambiguous situations. These associations are suggested to emerge from a generalization of parents’ own threat-vigilant information processing style to the child’s environment, potentially driven by parents’ perceptions of responsibility to protect their child (in addition to themselves) from aversive experiences.

### Threat Interpretations

Earlier empirical studies reviewed by Creswell and colleagues (2010) revealed significant links between parents’ interpretation of ambiguous situations involving themselves and their 4-to-10-year-old children (e.g., Lester et al., 2009), and between mothers’ threat/stress interpretations and expectations of their 10–11-year-old’s child threat/stress interpretations (Creswell & O’Connor, 2006). Parents’ threat and distress interpretations were measured with the adult version of the ambiguous scenarios questionnaire, whereas their expectations of child threat and distress interpretations were measured with the parent version in the earlier study by Creswell and O’Connor (2006). A later longitudinal study by Creswell and colleagues (2011) investigated the links between parents’ threat interpretations and expectations of their 5-to-9-year-old child’s threat interpretations using the same measure in a community sample in three successive yearly measurements. The results of this study partially support the idea of a link between parents’ interpretations and their expectations of their child’s interpretations: there were concurrent associations between parents’ distress cognitions and their expected child distress cognitions at all three-time points, whereas the link between parents’ own threat interpretations and their expectations of child threat interpretation was only significant at the third measurement. In turn, the findings revealed no significant longitudinal link between parents’ cognitions and their subsequent expectations of child distress/threat cognitions.

Another study addressed the link between parents’ self-referent and child-related threat interpretations, and negative expectations in a sample of 271 anxious and non-anxious mothers with 7-to-12-year-olds (Orchard et al., 2015). Mothers’ cognition, as well as their expectations of child cognition, were measured using ambiguous scenarios. Mothers additionally reported their expectations of child negative reactions, child perception of control, and performance in response to real-life challenges. The findings showed a positive significant association between mothers’ threat interpretations and their expected child threat interpretations across hypothetical and real-life situations. Moreover, maternal threat interpretations mediated the link between maternal anxiety disorder status and negative expectations of child coping in this study. This finding indicates that anxious parents’ negative expectations of child coping are shaped by their own threat interpretations.

Taken together, the findings on the links between parents’ own interpretations of threat and distress and their expectations of child interpretations are mixed (see Table 2 for an overview), as this concurrent link has not been observed consistently across the domains of threat and distress, across measurement points, and does not seem to hold prospectively. The heterogeneity of the samples and the age groups studied makes it difficult to infer whether this link changes across development, or as a function of parental and/or child anxiety status. Considering that parents reported their interpretation, as well as their expected child interpretation in questionnaires, it remains unclear to what extent this link is accounted for by measurement errors stemming from single-respondent bias, and demand characteristics.

**Table 2:** Studies testing the association between parents' threat/distress interpretations and their expectations of child threat/distress interpretations

Study(author last name, et al., year)	Parent *N Gender* (% female)	Youth Age M (SD, range)	Target Population	Youth Anxiety Symptom Measure	Parent Psychopathology	Parent Own Bias Measure	Parent's Expectations about Youth Bias Measure	Link Parents' Own-Expected Youth Bias	P-value
Creswell et al. (2011)	107 (96.3)	Not reported (not reported, 5–9)	Children selected based on parent-report of child anxiety to allow a normally distributed sample	Child report of anxiety in a 10-item cartoon adaptation of the anxiety items in the CBCL and ARBQ Parent report of child anxiety in a parallel 10-item questionnaire measure	Trait anxiety in STAI	Adult version of the Ambiguous Situations Questionnaire (ASQ-a)	Parent version of child ASQ (ASQ-pc)	*Cross-Sectional:* Threat: NS at T1 and T2, *b* = 0.32, *SE* = .15 at T3 Stress: *b* = 0.45, *SE* = .07 at T1, *b* = 0.48, *SE* = .09 at T2, and *b* = 0.08 *SE* = 0.4 at T3 *Longitudinal* Threat: NS, Stress:NS	*Cross-Sectional:* Threat: > .05 at T1 and T2, < .05 at T3 Stress: < .001 at T1 and T2, < .05 at T3 *Longitudinal* Threat: > .05 Stress > .05
Orchard et al. (2015)	271 (100)	9.9 (1.6, 7–12)	Clinically anxious children of parents with and without anxiety disorder	ADIS-IV-C/P SCAS-C/P	ADIS-IV Anxiety DASS-21	Adult version of the Ambiguous Situations Questionnaire (ASQ-a)	Parent version of child ASQ (ASQ-pc) In vivo challenge tasks: expectations	*r* = .35	< 0.02

Notes: M = Mean, SD = standard deviation, r: correlation, b: unstandardized estimate, SE: Standard Error, NS: Not Significant and Not Reported

### Attention to Threat

The studies on longitudinal and cross-sectional links between parents’ own cognitive bias and parents’ expectations of child cognitive bias have exclusively focused on threat interpretations, whereas it seems that no studies have yet investigated these links in attention. Thus, for the time being, it remains unclear whether parents hold expectations of their child’s threat-related attention, and whether these expectations can be assessed reliably. As self-report measures may not be suitable to capture these earlier more automatic associations, it will be first necessary to develop new computerized instruments that reliably capture the attention bias of parents, together with parents’ expectations of attention bias, to address this link.

There is, however, some empirical support for the idea that induced anxiety states can trigger attention bias to child-related words in parents. Cartwright-Hatton and colleagues investigated the effect of parental anxiety states on attention bias in a sample of 6-to-10-year-old children and their parents (Cartwright-Hatton et al., 2014). Parents were assigned to one of three conditions: Parents in the social anxiety condition were informed that they will have to give a presentation that would be recorded. Parents in the child anxiety condition were exposed to visual stimuli depicting potentially threatening situations for their child. Parents in the control condition were exposed to pictures of buildings. Following this manipulation, parents’ attention bias was measured in a visual probe task that included child threat-related versus neutral, or social threat versus neutral, word stimuli. The results revealed that parents in the social anxiety condition tended to show an attention bias towards social threat, whereas the attention bias of parents in the child anxiety condition concerned child-related threat. Note however that earlier work from Gallagher and Cartwright-Hatton (2009) did not reveal such specificity: researchers had shown that child-related attention bias can also be triggered in the social anxiety condition. Thus, it appears that induced parental state anxiety may lead to an attention bias towards child-related threats, although the links of this bias to parents’ general attention bias to threat, and expectations remain currently unclear.

### Do Parents’ Expectations of Child Threat Cognitions Relate to Their Child’s Actual Threat Cognitions?

In addition to the association between parents’ information biases in novel situations involving themselves and their child, the model by Creswell and colleagues (2010) incorporated a link from parents’ expectations/predictions of child threat cognitions to actual child threat cognitions. The findings on this additional link are addressed in this section.

#### Threat Interpretations

In an earlier longitudinal study, Creswell and colleagues investigated the concurrent and prospective correlations between maternal expectations of child interpretations and actual child interpretations among 10–11-year old children and mothers in two consecutive measurements in a period of six months (Creswell et al., 2006). Child interpretation bias was measured in response to ambiguous scenarios and included the dimensions of threat and distress in this study. Threat scores were based on the number of threatening interpretations, whereas the distress scores were based on the anticipated distress in response to ambiguous scenarios. Concerning the direct concurrent links between maternal expectations and actual child cognitions, the only significant positive association at T1 was between maternal expectations of threat and child distress cognitions, whereas no specific direct link in the dimension of threat or distress was found between maternal expectations and the actual child interpretation bias. In contrast, at T2, specific positive links were found between maternal expectations and child interpretation in the domains of threat and distress. Similar to T1, there was a positive association between parental expectations of threat interpretations and child distress cognitions at T2. Moreover, this study provided the first preliminary evidence for longitudinal, reciprocal associations: maternal expectations of child distress interpretations predicted change in child threat interpretations from T1 to T2. In turn, daughters’ (but not sons’) threat interpretations predicted change in maternal expectations of daughters’ threat interpretations from T1 to T2.

Creswell et al. (2011) further explored the idea that the direct link between child and parent cognitions is mediated by parental expectations of child cognition in a later longitudinal study in a community sample with three consecutive yearly measurements of parents’ expectations of child distress/threat cognitions and actual child distress/threat cognitions. No significant concurrent links were found between parents’ expectations of child threat/distress interpretations and actual child threat/distress interpretations in this study, except for a concurrent positive link in threat interpretations at T3. Moreover, parents’ expectations of child distress interpretations at T1 predicted actual child distress interpretations at T2, whereas no significant prospective link was found for threat interpretations. None of the direct links between parent and child cognition were significant, except for a negative prospective link between parent threat cognitions at T1 and child threat cognitions at T3. Thus, taken together, the findings do not seem to support the hypothesis of a direct link between child and parent cognitions mediated by parental expectations. This study however revealed that parents’ expectations of threat at T3 mediated the longitudinal link in child threat cognitions from T2 to T3. Thus, it seems that parents’ expectations may partially account for the change in 5-to-9-year-old children’s threat cognitions over time. Another study testing this concurrent link in a sample of older children (7-to-17-year-olds) with anxiety diagnosis using the adult and parent versions of ambiguous scenarios questionnaires reported a direct link between parents’ expectations of child threat interpretations and child self-reported interpretations (Blossom et al., 2013).

Taken together, the findings on the concurrent and prospective links between parental expectations of child threat cognitions and actual child threat cognitions reveal mixed findings. These associations were not consistently observed across measurement points, and across threat and distress interpretations in longitudinal studies (for an overview see Table 3). Although there is a pattern in the two prospective studies by Creswell and colleagues (2006, 2011) that suggest that the concurrent links may only hold in the latest measurement points, the range of the age groups studied, and the short intervals between measurement points make it difficult to make inferences on the developmental patterns. Furthermore, the heterogeneity of the samples studied makes it hard to distinguish how this link is moderated by child anxiety diagnoses/symptoms. Finally, the findings reveal no support for the idea that the direct link between parent and child threat cognitions would be mediated by parental expectations of child threat cognitions. In contrast, the findings from the two prospective studies by Creswell and colleagues (2006, 2011) provide support for the idea that change in child threat cognitions over time is mediated by parental expectations.

**Table 3:** Studies testing the association between parents' expectations on child threat/distress interpretations and actual child threat/distress interpretations

Study(author last name, et al., year)	Youth *N Gender* (% female)	Youth Age M (SD, range)	Target Population	Youth Anxiety Symptom Measure	Youth Bias Measure	Parent *N Gender* (% female)	Parent Psychopathology	Parent's Expectations about Youth Bias Measure	Link Parents' Expected Youth- Actual Youth Threat Bias	P-value
Creswell et al. (2011)	110 (57.3)	Not reported (not reported, 5–9)	Children selected based on parent-report of child anxiety to allow a normally distributed sample	Child report of anxiety in a 10-item cartoon adaptation of the anxiety items in the CBCL and ARBQ Parent report of child anxiety in a parallel 10-item questionnaire measure	Child version of the Ambiguous Situations Questionnaire (ASQ-c)	107 (96.3)	Trait anxiety in STAI	Parent version of child ASQ (ASQ-pc)	*Cross-Sectional:* Threat: NS at T1 and T2, *b* = 0.66, *SE* = .19 at T3 Distress: NS *Longitudinal* Threat: all NS Distress: all NS except parent expectation T1-child cognition T2 *b* = 0.52, *SE* = .26,	*Cross-Sectional:* Threat: > .05 at T1 and T2, < .001 at T3 Distress: > .05 *Longitudinal* Threat: > .05 Distress: all > .05, except parent exceptation T1-child cognition < .05
Blossom et al. (2013)	488 (49.6)	10.7 (2.8, 7–17)	Children selected based on the presence of a current anxiety disorder	ADIS-IV-C/P SCARED-C/P	Child version of the Ambiguous Situations Questionnaire (ASQ-c)	Not reported	trait anxiety in STAI	Parent version of child ASQ (ASQ-pc) In vivo challenge tasks: expectations	*r* = .21	< .01

*M* = Mean, *SD* = standard deviation, *r:* correlation*,* b*:* unstandardized estimate*,* SE*:* Standard Error*,* NS: Not Significant and Not Reported

#### Attention to Threat

The findings on the link between parents’ expectations of child threat cognitions and actual child threat cognitions come from studies that exclusively focused on later stages of information processing, such as interpretation and perceptions. These associations have not been tested in attention. Thus, testing the associations between parents’ expectations of child attention, and actual child attention bias towards threat, relies on future innovative research designs that would allow for a reliable measurement of both components.

#### Child Perceptions of Distress, Coping, and Performance

The links between parents’ expectations and child perceptions of child distress, coping, and performance were investigated in a sample of 6-to-14-year-olds and their anxious (versus non-anxious) mothers (Becker & Ginsburg, 2011). Children were asked to give a speech about themselves following a 5-min planning phase where mothers and children worked together to prepare the speech. Prior to the speech, parents reported their expectations of their child’s distress, coping, and performance. Following the speech, children reported their anxiety, as well as their self-evaluations of distress, coping, and performance in this situation. The findings revealed no significant direct links between parents’ expectations and child perceptions in the domains of distress, coping, or performance in this study.

## Summary and Future Directions

Studies on the links between parents’ own cognitive bias and parents’ expectations of child cognitive bias, as well as between parents’ expectations of child cognitive bias and the actual child cognitive bias, have predominantly focused on threat/distress interpretations. No studies have yet investigated these links at the level of attention. Thus, the links between anxious parents’ attentional vigilance to the ambiguous aspects of their own and their child’s environments, and between parents’ attentional vigilance and expectations of child attention to threat are related to actual child attention await further investigation. The only study focusing on the perceptions of distress, coping, and performance revealed no significant link between parental expectations of child cognition and actual child cognition. The findings at the level of interpretations are mixed, as these links were not consistently observed over time, across threat and distress interpretations, across the age ranges studied, or across clinical and community samples. Although there is a certain level of consistency in the measurement of threat interpretations using ambiguous scenarios, the heterogeneity of the samples studied in terms of age, and presence of psychopathology in the parent or offspring, do not allow a clear conclusion regarding moderation by child or parent anxiety diagnoses/symptoms. Thus, the idea that anxious parents’ cognition shapes their expectations of child interpretations of threat and distress, and that this can turn into a self-fulfilling prophecy impacting the child’s actual interpretations remain to be further investigated. Future prospective studies investigating these links in developmentally sensitive designs that incorporate community and clinical samples of parents (and/or children) would allow the study of change and stability of these associations over time.

## Is a Parents’ Information Processing Style Associated with Particular Parental Behaviors?

The available evidence indicates that anxious versus non-anxious parents are more likely to perceive ambiguous situations involving themselves or the child as more threatening, and less controllable, and hold more negative expectations of their own and their child’s experience of distress and threat in these situations (Orchard et al., 2015). Thus, anxious parents tend to overestimate the likelihood of potential threat in ambiguous situations involving their child and underestimate their child’s coping skills as compared to non-anxious parents. Creswell and colleagues (2010) proposed that anxious parents’ prioritized processing of potential threats in their own and their child’s environment, and negative expectations of their own and their child’s coping and control may, in turn, increase their likelihood to engage in parenting practices that aim to reduce their child’s (and own) negative experiences and enhance control in these situations. They suggest that these biases may enhance the parents’ tendency to promote avoidance of ambiguity, reduce their ability to encourage approach to ambiguous stimuli/situations, and grant autonomy to their child’s explorations. This combination leads to a more involved, controlling, and protective parenting style. In line with this proposal, there is meta-analytic evidence for a small association (*d* = 0.25, van der Bruggen et al., 2008) between parents’ anxiety and parental autonomy granting and control, whereas no significant link was shown between parental anxiety and parental control alone. Much less is known, however, on the link between parents’ cognitive biases and parenting dimensions of autonomy granting and control.

### Lack of Parental Autonomy Granting

#### Threat Interpretations

An earlier experimental study by Creswell and colleagues illustrates how negative expectations that parents have may impact their parenting (Creswell et al., 2008). A sample of primary caregivers (of 7-to-11-year-olds) was informed that their child would complete a difficult anagram task. Prior to the task, parents’ expectations of the child’s performance were experimentally manipulated via verbal information. Parents in one group were informed that the child is likely to enjoy the task, whereas parents in the other group were told that the child may struggle with the task and may find it upsetting. This information was sufficient to create a change in parents’ involvement during the task: Parents who heard negative versus positive expectations of their child’s performance showed greater involvement. Thus, induced negative expectations can trigger enhanced involvement in parents, which can be seen as an attempt to control the situation and the child anticipated distress reactions. This appears to be the only study that investigated this association between anxious parents’ information processing and their parenting practices.

The link between parental cognitions and parenting has been, however, addressed in a separate line of literature investigating parents’ cognition and parenting in the context of the dynamic exchanges in parent–child interactions in early development by focusing on the construct of maternal accuracy of child distress to novelty (Kiel & Buss, 2010, 2011; Kiel et al., 2021). Parental accuracy refers to parents’ ability to anticipate their child’s avoidant and negative reactions in response to novel/ambiguous situations. Thus, parental expectations of child reactions are considered not in isolation, but in relation to observed child reactions in this line of literature. To study parental accuracy, parents were asked to report their expectations for their child’s reactions to novelty (e.g., approach, play, wariness) in response to some ambiguous stimuli (e.g., clowns, puppets, strangers). Their child was later exposed to these stimuli in standardized temperament tasks. Parenting dimensions included (observed or reported) overprotective and oversolicitous parenting, comforting the child in a way that promotes avoidance and withdrawal from the novelty. The findings from these studies have consistently revealed that when parents accurately anticipate their temperamentally fearful child’s distress, they are more likely to engage in over-solicitous/overprotective parenting (Kiel & Buss, 2010, 2011). This link, however, only held when parents make highly accurate predictions, and when children were temperamentally fearful.

In a more recent study where Kiel and colleagues investigated this link in a prospective design (Kiel et al., 2021), it was found that the continuity of parental accuracy between toddlerhood and kindergarten years occurs through an indirect path: Parents of temperamentally fearful children who show high accuracy in their expectations of child reactions show overprotective parenting, that in turn is related to children’s social withdrawal in kindergarten, that in turn predicts parents’ accuracy in kindergarten years. These findings point to the dynamic and bidirectional influences that shape the link between parental expectations and parenting and highlight the moderating role of fearful temperament in this link.

#### Attention to Threat

Although the bidirectional causal link between anxiety and attention bias has received consistent empirical support in earlier adult studies (Van Bockstaele et al., 2014), no evidence is available on the links between parents’ attention bias and autonomy granting. Thus, we do not know if anxious parents, who may be more likely to attend to threats in their own and their child’s environment, and may in turn expect their child to react similarly, would be more likely to engage in protective or controlling parenting.

### Fear-Enhancing Parenting Behaviors: Modeling and Threat Information Transmission

According to Field and Lester (2010), each confrontation with a novel/potentially ambiguous situation in everyday life acts as a single-trial parental cognitive bias training, where the parents disambiguate the situation for their child, either in a safe or threatening way with their reactions. The model by Creswell and colleagues (2010) suggests that anxious parents who are, by definition more likely to experience high levels of anxiety and threat-related information processing bias in these situations would be more likely to model anxious behavior and to verbally communicate higher levels of anxiety and threat information to their child. Although there is empirical evidence showing that anxious parents show higher levels of non-verbal anxiety displays (e.g., Aktar et al., 2013; Murray et al., 2008) and a higher likelihood to verbally communicate threat/anxiety (Murray et al., 2014; Percy et al., 2016), a lot less is known regarding the associations between fear-enhancing parenting behaviors and parents’ cognition.

#### Threat Interpretations

The few studies addressing the question of how parents’ threat perception of ambiguous stimuli may affect their fear-enhancing parenting and transmission of cognitive bias in community samples relied on the manipulation of parental threat perceptions about a novel stimulus via verbal information. Muris and colleagues (Muris et al., 2010) provided parents with positive, negative, or ambiguous information about unknown animals. Next, parents discussed a number of open-ended vignettes with their 8-to-13-year-old children. They were instructed to talk about hypothetical confrontations with these animals, and about what would happen in these confrontations. Parents who received negative (versus positive) information generated narratives that included more threat information, thus verbally transmitting a threat interpretation bias to the offspring. When the information about the animal was ambiguous, the number of threat narratives depended on parents’ trait anxiety levels, with more anxious parents providing more threat information about the animals paired with ambiguous information. In a later experiment, Remmerswaal and colleagues (Remmerswaal et al., 2013) replicated these findings in a sample of 8-to-12-year-olds and their mothers using real novel animals. Mothers were provided with positive or negative information about the animals and were told that their children would confront the animals. They were then instructed to prepare their child for this confrontation. The findings revealed that parents provided more negative statements about the animals when they received negative information. The findings from these studies reveal that threat information transmission from researchers to parents may alter how parents transmit threat information to their offspring, enhancing the potential threat value of ambiguous stimuli.

A related line of research focuses on the associations between parents’ (social) anxiety/worry and fear-enhancing parenting behaviors at the start of schooling as a real-life ambiguous situation. The findings reveal that parental social anxiety or worry alters parents’ verbal information in a way that resembles the negative information manipulation by the two experimental studies (Muris et al., 2010; Remmerswaal et al., 2013). Parents with social anxiety disorder were more likely to verbally transmit threat information to their children in their narratives about the situation (Murray et al., 2014). Likewise, healthy parents reporting higher levels of worry about their child starting school have more negative narratives that include verbal threat information (Pass et al., 2017).

#### Attention to Threat

The associations of the fear-enhancing parenting behaviors with the bias in parents’ attention have not yet been addressed. Thus, the idea that anxious parents who show a threat-related attention bias would be more likely to engage in fear-enhancing parenting awaits investigation in future studies among samples of clinically anxious versus non-anxious parents.

#### Confirmation of Threat

Another study (Remmerswaal et al., 2010) investigated the effect of positive and negative (versus neutral) information about novel animals on parents’ biased processing at a different step of information processing (i.e., confirmation bias). Parents who received negative information about a novel animal were shown to display a confirmation bias towards threat: they were less likely to falsify threat information to their 9-to-12-year-old about the animal paired with negative versus positive information.

## Summary and Future Directions

Creswell et al. (2010) proposed that anxious parents’ information processing may impact their parenting practices during their child’s confrontations with ambiguous situations. Note however that no studies seem to have captured this association between anxious parents’ information processing and their parenting practices since this model was proposed. Thus, our knowledge is limited to the earlier empirical evidence of a causal link between parental negative expectations and involvement among parents of 7-to-11-year-olds (Creswell et al., 2008). Hence, the nature and the direction of this link between parental autonomy granting and parental cognitions and expectations remain to be further explored across stages of information processing. The lack of empirical studies at the level of parents’ threat relevant attention in relation to their parenting marks an important direction to be considered in future studies. An alternative line of research by Kiel and colleagues (Kiel & Buss, 2010, 2011; Kiel et al., 2021) revealed that the accuracy of parental predictions on child behavior relates to overinvolved/oversolicitous parenting only when children show a fearful temperament, and when parents can make accurate predictions. These findings emphasize the importance of considering parental expectations of child threat bias in future designs that include child anxiety dispositions, such as fearful temperament. Finally, it is important to note that the operationalizations of parenting dimensions differed across the limited number of studies investigating this link, including parental overcontrol, involvement, and oversolicitous/overprotective parenting.

Concerning the fear-enhancing parenting behaviors, no evidence is yet available on the associations of parental modeling of anxiety with the biases in parents’ information processing at the stages of attention and interpretation. The available evidence of the links of verbal threat transmission with parents’ cognitive bias reveals that threat information may impact parents’ narratives in a way that exacerbate the threat value of ambiguous stimuli: parents who received negative information about a novel stimulus were shown to transmit a higher number of threat interpretations and to display a confirmation bias towards the stimulus. There is also support for the idea that parents who experience higher levels of anxiety and worry are more likely to verbally induce a cognitive bias towards ambiguous situations via anxiety-enhancing parenting behaviors. The links between fear-enhancing parenting practices and parents’ information processing remain to be investigated.

## Are Parental Behaviors Associated with How Children Process Information?

The model on intergenerational transmission of information processing (Creswell et al., 2010) proposes that fear-enhancing parenting behaviors and reduced parental autonomy granting during child confrontations with novel situations convey to the offspring the idea that these situations are threatening and not controllable. Thus, repeated exposure to parents’ anxiety-enhancing parenting and lack of autonomy granting in these situations may contribute to parents’ biasing of the child cognitive resources towards threat, triggering anxious information biases and anxiety in children (for an overview of parental pathways to child anxious cognitions, see Emerson et al., 2019).

### Lack of Parental Autonomy Granting

#### Threat Interpretations

In an earlier study, Barrett and colleagues examined threat interpretations and possible action plans of 7-to-14-year-old anxious (versus non-anxious) children measured before and after they engaged in a family discussion of these scenarios (Barrett et al., 1996). Anxious children had initially higher levels of threat interpretations and were more likely to choose avoidant solutions than non-anxious children. Following discussion with parents who encouraged stronger threat interpretations and more avoidant strategies, there was an increase in anxious children’s avoidant solutions to ambiguous scenarios. In a more recent study on the effect of family discussions on child threat interpretations, Sicouri and colleagues have provided partial support to these findings (Sicouri et al., 2017). They measured child threat interpretations and avoidant solutions before and following family discussions in a sample of 8-to-13-year-old children with anxiety (and/or asthma versus controls) and their parents. Anxious children (with and without asthma) were more likely to adopt avoidant solutions to ambiguous scenarios, including asthma-related themes, although they did not show stronger general threat interpretations than non-anxious children prior to the family discussion. Following the family discussion, children with anxiety but without asthma displayed increased levels of avoidant solutions to both anxiety and asthma-related scenarios, whereas children with anxiety and asthma had more avoidant solutions specifically in response to asthma-related scenarios. These findings, together with the earlier findings from (Barrett et al., 1996), support the idea that parents who encourage more threat interpretations and avoidant responses to ambiguity in family discussions may enhance children’s tendency to provide avoidant solutions to imagined ambiguous situations.

Another study has specifically focused on the dimension of parental overcontrol in relation to child interpretation bias in a sample of 7-to-12-year-old children of parents with anxiety disorders (Affrunti & Ginsburg, 2012). This study addressed the interrelations between child anxiety and threat interpretations and parental anxiety and overcontrol. Child threat interpretations were measured using ambiguous stories. Parental overcontrol and child anxiety were measured using child reports, and parental anxiety was assessed with parent reports. The findings revealed a significant positive association between parental overcontrol and child threat interpretations: Thus, children of parents with higher levels of overcontrol had stronger threat interpretations. Moreover, child interpretation bias partially mediated the link between parental overcontrol and child anxiety, and fully mediated the link between parent and child anxiety in this study. These findings suggest that overcontrolling behavior in parents with anxiety disorders may lead to child anxiety by triggering stronger threat interpretations. Moreover, the findings of this study provide preliminary support for the idea that the link between parental and child anxiety may be fully accounted for by child threat interpretations of ambiguous scenarios.

#### Attention to Threat

The link between reduced parental autonomy granting and child attention bias to threat was explored in an earlier study in 6-to-14-year-old children and their mothers (Perez-Olivas et al., 2008). Child attention bias was measured in a visual search task with angry (versus neutral and happy) faces. Maternal overinvolvement was assessed in a phone interview where the mothers were asked to talk about their feelings and attitudes about their child. Overinvolvement in this study included emotional displays (for example crying during the call), and parental reports of overprotective/self-sacrificing behavior. Mothers and children also reported child separation anxiety in questionnaires. The findings revealed a direct link between maternal overinvolvement and child attention bias to angry faces. Moreover, child attention bias mediated the link between parental overinvolvement and child separation anxiety in this study, suggesting that the link between overinvolved parenting and child anxiety may emerge indirectly through this cognitive path.

A more recent study addressed this mediation by focusing on parental control in a sample of 6-to-18-year-old clinic-referred children and their mothers (Bose et al., 2019). Child attention bias was measured in a dot-probe task with angry (versus neutral) faces, whereas maternal control, and child anxiety severity were assessed using mother and child reports. The construct of maternal control included overprotective, manipulative, intrusive, and guilt-inducing parenting. The direct links between child attention bias and anxiety severity, and between child attention bias and maternal control were not significant in this study, whereas the direct link between maternal control and child anxiety severity was significant. Thus, these findings do not reveal a direct link between maternal control and child attention bias and do not support the idea that the link between parental control and child anxiety is mediated by child threat-related attention bias.

Inconsistent findings across these two correlational studies can be accounted for by differences in the operationalization and measurement of autonomy granting (parental overinvolvement versus overcontrol), or in the measurement of child attention. No studies, however, have yet investigated the links between parenting and child attention in experimental designs. Thus, the idea that decreased levels of parental autonomy granting during child confrontations with novel situations trigger child attention bias to threat remains to be investigated.

#### Child Perceptions of Distress, Coping, and Performance

The link between parental overcontrol and child perceptions of distress, coping, and performance was investigated in a sample of 6-to-14-year-olds and their anxious (versus non-anxious) mothers in the study by Becker and colleagues (Becker & Ginsburg, 2011). Parental overcontrol was observed during the preparation phase where the parent helped their child to prepare for a speech performance and included overinvolved, directive, and intrusive behaviors, as well as unsolicited support. Following the performance, children reported their perceptions of distress, coping, and performance. Children of mothers who showed more overcontrolling parenting during the preparation phase had stronger perceptions of distress, and evaluated their performance more negatively. There was no link between overcontrol and child coping perceptions in this study. In another study, parents were trained to show controlling versus autonomy granting parenting prior to a speech task by their 4–5-year-old-child (Thirlwall & Creswell, 2010). Children reported more negative expectations/predictions of their performance prior to the speech in the controlling versus autonomy granting condition, whereas their perceptions of performance following the speech did not seem to differ between conditions. Taken together, the findings suggest that lower levels of parental autonomy granting may enhance child negative predictions/evaluations of their stress and performance before and after stressful situations.

### Fear-Enhancing Parenting Behaviors: Modeling and Threat Information Transmission

Earlier studies focusing on the effects of fear-enhancing parenting behaviors (modeling or threat information transmission) on child reactions studied the effect of parental modeling and threat information transmission on child behavioral reactions in the lab. Despite consistent evidence of a significant effect of anxious parents’ modeling of non-verbal anxiety displays in novel situations on child observed avoidance as early as in infancy (Aktar et al., 2013; de Rosnay et al., 2006; Murray et al., 2008), relatively less is known regarding how parents’ verbal and non-verbal anxious responses during confrontations with novelty shapes child threat relevant information processing. The few experimental studies looking at the effect of modeling and threat information transmission have focused on a later stage of information processing (i.e., child fear beliefs). More recently, questionnaire measures of fear-enhancing parenting behaviors were incorporated into the study of associations between parenting and child confirmation and interpretation bias.

#### Threat Interpretations, and Confirmation of Threat

Fliek and colleagues (Fliek et al., 2017) investigated the cross-sectional links between child cognitive biases (i.e., threat interpretation and confirmation), anxiety, and fear-enhancing parenting behaviors of mothers and/or fathers in a community sample of 258 7-to-12-year-old children. The researchers designed a questionnaire to measure fear-enhancing parenting behaviors (modeling and threat information transmission) based on child and parent reports. Child threat interpretations were measured with the ambiguous stories task and child confirmation bias was measured in an information search task. In this task children could choose between a positive or negative question to gain information about hypothetical ambiguous situations. Children with higher levels of anxiety showed stronger threat interpretations and a more negative confirmation bias, and reported that their parents show higher levels of fear-enhancing parenting behavior. Moreover, the link between parental threat information and child anxiety was fully mediated by child cognitive bias (interpretation and confirmation bias). Child interpretation (but not confirmation bias) also partially mediated the relationship between modeling and child anxiety. Taken together, the findings of this study provide evidence of a direct link between fear-enhancing parental behaviors and child cognition at the levels of interpretation and confirmation biases, and of a mediating role of child cognitions in the link between parental fear-enhancing parenting and child anxiety.

In a follow-up longitudinal study of the associations between parenting, child cognitive bias, and child anxiety (at 3-time points over a year), Fliek and colleagues (Fliek et al., 2019) tested three alternative models: The first model linked fear-enhancing parenting both to cognitive (interpretation and confirmation) biases and anxiety symptoms, whereas the second (reverse) model tested the possibility that child cognitive bias and anxiety symptoms lead to fear-enhancing parenting. A third model tested bidirectional associations between fear-enhancing parenting and child cognitive bias and anxiety symptoms. The best fit was observed with the second reverse model, raising the possibility that higher levels of child cognitive bias and anxiety symptoms lead to fear-enhancing behavior in parents. The study also revealed evidence of longitudinal bidirectional associations between child anxiety and cognitive biases. Thus, the idea that the effects of anxiety-enhancing parenting on child anxiety are mediated by child cognitive biases was not supported.

#### Attention to Threat

Our review revealed no studies on the associations between fear-enhancing parenting behaviors and child attention to threat. Thus, the idea that fear-enhancing parenting behavior is related to vigilant processing of threat remains to be investigated at the level of attention bias in correlational and experimental designs.

#### Fear Beliefs

An experimental study by Dunne and Askew (2013) tested the effect of modeling (or vicarious learning) from parents on the acquisition of fear beliefs in 6-to-10-year-old children. In a computer task, children were presented with pictures of novel animals paired with positive or fearful facial expressions of parents and strangers (Dunne & Askew, 2013). Children reported higher fear beliefs to the animals paired with their parents’ (or a stranger’s) fearful versus happy facial expressions. Although the findings of this study provide some support for the idea that parental expressions of fear in response to novel animals may act as a threat signal and trigger cognitive bias in children, it remains unclear how the effect of parental modeling on child cognition unfolds in real-life. This question awaits to be investigated in experimental designs with higher ecological validity.

Experimental studies on the effect of threat information transmission from parents on child cognition consistently reveal a significant effect of parents’ verbal threat information on child fear cognitions (i.e., fear beliefs). For example, providing parents with negative versus positive, or ambiguous information about unknown animals triggered higher levels of parental threat narratives about the animals paired with negative versus positive information (Muris et al., 2010). In turn, higher levels of parental threat narratives triggered higher levels of fear beliefs in children. When the information was ambiguous, child fear beliefs depended on the parents’ trait anxiety: more anxious parents communicated stronger threat about the animals, which in turn was related to higher levels of child fear beliefs. In contrast, in the study by Remmerswaal and colleagues (2013) who provided mothers with negative versus positive information, more negative statements from the parent about the animal paired with negative information did not trigger higher fear beliefs in 8-to-12-year-olds. They did, however, trigger a behavioral reluctance to approach the novel animals. Thus, although the findings provide some support for a significant effect of verbal threat information on child fear beliefs, it remains to be further investigated whether the effect of parental verbal information on child behavioral reactions to novel stimuli is mediated by alterations in child threat-related cognition, including fear beliefs.

#### Child Perceptions of Distress, Coping, and Performance

Becker and Ginsburg (2011) investigated the link between observed fear-enhancing parenting behaviors and 6-to-14-year-olds’ perceptions of distress, coping, and performance following a speech task. Observed maternal anxiety included non-verbal facial and bodily expressions of anxiety (such as lip-biting, fidgeting, or a worried facial expression) as well as verbal comments that convey anxiety during the preparation phase. Mothers’ anxious behavior before the performance did not predict child perceptions of distress or performance, while it predicted more negative coping perceptions.

In an experimental study focusing on the effect of fear-enhancing parenting behaviors prior to a spelling task on child cognition, parents were trained to express verbal and non-verbal anxious or non-anxious reactions to their 8-to-12-year-old child prior to a spelling test (Burstein & Ginsburg, 2010). Each child completed two spelling tests following parental anxious versus non-anxious reactions and rated their perceptions on the difficulty of the test and their performance in a questionnaire. The findings revealed that children who were exposed to fear-enhancing parenting in the form of verbal and non-verbal anxiety expressions held more negative cognitions about the test, and the effect seemed to be more pronounced with fathers. Taken together, these studies provide some support for the idea that parents’ fear-enhancing behaviors may lead to child negative perceptions about stressful situations.

## Summary and Future Directions

The few studies that addressed the links between observed parenting and child information processing in middle to late childhood years (7-to-14-years) reveal that parents may contribute to the strengthening of initial anxious dispositions in child information processing by encouraging threat interpretations and avoidant solutions to ambiguous scenarios during family discussions. There is also preliminary support for the idea that children whose parents show more controlling parenting show higher levels of threat interpretations. The limited research on the direct link between parental autonomy granting and child attention bias has revealed mixed findings that may be explained by the differences in the parenting constructs studied, as well as in the measurement of attention bias. The reviewed studies also show some support for a link between parental autonomy granting and child negative predictions/perceptions of distress levels and performance in stressful situations.

Concerning fear-enhancing parenting, the evidence summarized in this section reveals a significant effect of parental fear-enhancing parenting behaviors (modeling and transmission of verbal threat information) on child cognitive bias towards novel stimuli at the level of fear beliefs. More recent evidence relying on child and parent reports of fear-enhancing parenting revealed a direct link between fear-enhancing parenting and child cognitive biases at the stages of interpretation and confirmation of threat. Moreover, this line of research brings important insights on the mediational role of child cognitive bias in the link between fear-enhancing parenting and child anxiety. This work also highlights the bidirectional nature of the links between child cognitive bias and fear-enhancing parenting. For future research, it will be important to validate questionnaire measures of fear-enhancing parenting in studies that incorporate observations of this construct. There is also some evidence suggesting that maternal fear-enhancing parenting in the form of verbal and non-verbal anxiety expressions prior to a stressful task may be related to child negative perceptions of coping. Future studies will need to further investigate the specificity of this effect across the domains of distress, coping and performance before and after the task. Finally, the lack of empirical studies on the associations of fear-enhancing parenting with child attention marks another important direction for future research.

## Discussion

### An Updated Conceptual Model

**Fig. 2.**
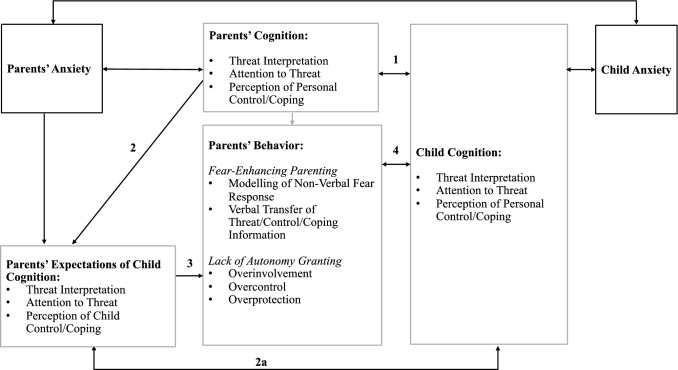
The cognitive-behavioral model of intergenerational transmission of anxious information processing biases: an updated conceptual model

This theoretical review collated recent empirical work testing the four core hypotheses of the model on the intergenerational transmission of anxious information processing biases by Creswell and colleagues (2010). The work reviewed and summarized in the sections above provides some general insight into the nature and direction of the hypothesized associations and helps to identify knowledge gaps and limitations in this line of literature. In the light of this insight, an updated conceptual model on the intergenerational transmission of anxious information processing biases is proposed with the following adjustments (presented in Fig. 2):

First, the construct of (parental/child) cognition was limited to threat interpretations in the original model as there was little or no empirical work at the time testing the hypothesized links at other stages of information processing. Although recent studies have increasingly started to address some of the hypothesized links in attention (e.g., the similarities in parent and child cognition, see Sect. 1.2), attention is still among the most under-studied areas in this line of work. Although it is too early to assume that the hypothesized associations hold across attention and interpretation, the updated model incorporates attention to the construct of cognition to stimulate further research in this domain. Furthermore, despite their first core hypothesis on the similarities between cognitions of parents and children, the direct link between parents’ and child cognitions was not included in the original figure by Creswell et al., (2010, Fig. 1), possibly to signal a full mediation by parents’ expectations and behavior. In the absence of evidence for full mediation, this link was incorporated as a direct association in the updated model (1 in Fig. 2).

Moreover, while the original model clustered parents’ behaviors into two overlapping constructs, parents’ behavior appears in the updated model as a single dimension that consists of two components: fear-enhancing parenting behaviors and parental autonomy granting. Fear-enhancing parenting in the updated model includes modeling of non-verbal fear response and verbal transfer of threat/control/coping information. For a clearer distinction between non-verbal and verbal pathways to fear transmission, the use of the term modeling was limited to the non-verbal observational/vicarious learning pathway, and the use of the term information transfer of threat was limited to the verbal transmission. The updated model also incorporated the specific autonomy granting parenting constructs (i.e., parental overinvolvement, overcontrol, and overprotection).

Another adjustment concerns the directionality of the postulated associations between parental dimensions and child cognition (associations 1, 2a, and 4). These were unidirectional in the original model by Creswell and colleagues (2010, Fig. 1), predominantly highlighting the parent-to-child direction of effects. However, summarized evidence repeatedly hints at the potential child-to-parent influences (Fliek et al., 2019; Creswell & O’Connor, 2006; Creswell et al., 2010). The proposed child-parent associations are likely to be dynamic and bidirectional in nature. Thus, it is important to acknowledge that this is rather a two-way street, where the parental dimensions, child anxious information processing and anxiety may feed into each other, increasing the anxiety experiences and anxious information processing in the family. The child-to-parent direction of the links has been incorporated in the updated model: the links between parental cognition and child cognition (1 in Fig. 2), between parental behavior and child cognition (4 in Fig. 2), between parents’ expectations of child cognition and actual child cognitions (2a in Fig. 2), and between parent and child anxiety are bidirectional (Fig. 2).

Moreover, although the causal links between anxiety and information processing of threat in parents and children are at the core of the model on the intergenerational transmission of cognitive biases (Creswell et al., 2010), the bidirectional links between anxiety and cognition in parents and children were not part of the core hypotheses of the original model. The available empirical work on the hypothesized associations provides little insight into how the proposed links may change as a function of parental or child anxiety status. The bidirectional links between anxiety and cognition on the side of parents and children were incorporated into this updated model to encourage their inclusion in future research. The bidirectionality of the anxiety-cognition link poses an inherent challenge to the assumption of cognitive biases fully mediating the link between anxiety in parents and children. It will be important to capture the real complexity of this net of associations between anxiety, cognitive biases, and parental influences in future research.

This updated model awaits to be tested in future designs that address the general limitations of the current empirical studies, summarized in the next section. As a final note, it is important to acknowledge that the direct and indirect associations between parent and child cognitive biases appear more complex than depicted in this updated model. For example, the model does not incorporate the developmental influences, or moderation of the hypothesized links by parent and child characteristics (other than anxiety) on parenting and child cognitions. In addition, the earlier evidence reveals the possibility that parental bias at earlier stages of information processing may impact the bias at later stages of child information processing, whereas the interrelations between different stages of processing are not depicted in the updated model. Developmentally sensitive longitudinal designs that entail bidirectional influences at different stages of information processing in parents and children, together with parent and child anxiety, will be crucial for a better understanding of the shared information processing styles in parents and children.

### General Limitations and Directions for Future Research

General limitations characterizing the empirical work addressing the model by Creswell and colleagues (2010) are outlined below to set the main directions for future work.

1. Developmental time frames and mechanisms. The available evidence does not allow any conclusions on the developmental time frames of the hypothesized associations, as the majority of empirical work relied on cross-sectional correlational designs. Future research should adopt a developmental approach to investigating the hypothesized links by testing the associations in longitudinal designs measuring the constructs of interest. This would provide insight into the timeline and the direction of the hypothesized associations. In a related vein, the developmental mechanisms explaining the hypothesized links remain unknown. Future longitudinal studies should incorporate genetic and environmental mechanisms that may account for the emergence and/or the maintenance of the hypothesized links.
2. Threat bias across different stages of information processing. The available empirical work on the hypothesized associations predominantly relies on the investigation of threat interpretations, whereas biases in parents and child information processing have not been simultaneously addressed across different stages of information processing. Future research should consider testing these associations in longitudinal studies that integrate the assessment of child and parent cognitions in two or more stages of information processing.
3. Measurement of threat bias. The methods and instruments used in the measurement of threat-related bias in parents and children not only showed considerable heterogeneity across studies, but were also limited with respect to reliability and ecological validity, and prone to measurement errors (such as single-respondent bias and demand characteristics). Recent advances in mobile eye-tracking technologies provide a unique opportunity to capture child cognitive biases in real-life situations while integrating the measurement of cognitive biases across various stages of information processing (e.g., Allen et al., 2020). Future studies aiming to address the hypothesized links should move towards these more reliable, and ecologically valid measurements of cognitive biases in parents and children.
4. Moderation by parent and/or child anxiety diagnosis. Given the heterogeneity of the samples studied in earlier work, the available evidence does not allow an understanding of whether the hypothesized links differ as a function of child or parent anxiety diagnostic status. Future longitudinal designs must simultaneously include community and clinical samples of parents (and/or children) to investigate a potential moderation of the hypothesized associations by parental or child anxiety diagnosis/symptoms.
5. Parental influences. The available evidence does not answer the question of whether the hypothesized links between parental cognitions and parenting and child cognition differ across mothers and fathers, as most studies focused on mothers. In addition to the differential parental influences, the joint effect of maternal and paternal influences in two-parent families is an important area for future research.

### Clinical Implications

The postulated associations raise the question of whether training parents’ cognitive bias can indirectly contribute to child anxiety interventions. A study that targeted mothers’ child-related cognitions using an interpretation modification program revealed that the beneficial effects of such parental training do not always generalize to child cognition or behavior (Benoit, 2013). Thus, it seems that the generalization of the change in parental interpretation bias to child cognition and behavior will be an important future challenge to address. The idea that parental interventions targeting a reduction in fear-enhancing and an increase in autonomy granting parenting may benefit the child by reducing anxious cognitive bias and anxiety will be another important aspect to address in future studies.

## Data Availability

N/A.

## Code Availability

N/A.
